# Gender aspects and influence of hormones on bronchial asthma – Secondary publication and update

**DOI:** 10.1186/s40413-017-0177-9

**Published:** 2017-12-27

**Authors:** Iris Koper, Karin Hufnagl, Rainer Ehmann

**Affiliations:** 1 Department of Internal Medicine/Pneumology, Sana Kliniken Ostholstein, Clinics Oldenburg, Oldenburg, Germany; 2 Comparative Medicine, The Interuniversity Messerli Research Institute of the University of Veterinary Medicine Vienna, Medical University Vienna and University Vienna, Austria Veterinärplatz 1, 1210 Vienna, Austria; 3 Severe Asthma Center, Ambulante Pneumologie mit Allergiezentrum (BAG), Rotebühlplatz 19, 70178 Stuttgart, Germany

**Keywords:** Asthma, Contraceptives, Gender, Sex hormone, Menopause, Pregnancy, Sex, Smoking

## Abstract

There is good evidence for gender-specific differences in asthma regarding all affected areas, from intra- to extra-cellular mediators to the whole organ structure und functioning of the lung. These result from complex, in parts synergistic, in other parts opposing, effects — especially of female sex hormones, and rather protective effects of male hormones against asthma, which include effects on the cellular immune system. Additionally, there are gender differences of sociocultural origin, regarding presentation, doctor’s diagnosis and treatment of asthma symptoms, as well as the undertaken coping strategies concerning the female or male patient’s complaints. Taking into account gender-specific differences in asthma would contribute to improved individual diagnosis and therapies.

## Background

Asthma is a common chronic inflammatory disease of the airways [1], which leads to variable or even persistent airflow limitation. The main symptoms are dyspnoea (shortness of breath), wheezing, chronic cough and chest tightness. The prevalence for asthma in humans varies worldwide affecting 1 to 18% of any investigated population [1]. Women are more often affected by asthma. The mechanisms underlying the gender differences in asthma prevalence are still under investigation but refer mostly to hormonal differences and differences in lung capacity [2].

In this review article we will highlight the role of sex hormones in asthma pathogenesis using data from epidemiological, clinical and animal model studies. The basis of our systematic and thorough literature search is listed in Table 1 with keywords and selection criteria.

**Table 1:** Database, keywords and selection criteria for literature search on gender aspects in bronchial asthma

Systematic Literature Search^a^		
Database	Biosis, Embase, International Pharmaceutical Abstracts, Medline	
Selection Criteria	Asthma Gender Epidemiology	from 2003
	Asthma Gender Pathophysiology	
	Asthma Gender Symptoms	
	Asthma Gender Diagnostics	
	Asthma Gender Therapy	
	Asthma Sex Hormones	1995–2003
	Role of IgE in Menopausal Asthma	
	Therapeutic Response to Omalizumab and Gender-specific Differences	

^a^Update for secondary publication until 2017

## Epidemiology of bronchial asthma

While bronchial asthma affects about 300 million people worldwide, asthma incidence and severity are higher in women than in men, and highest in women between the 4th and 6th decade. During childhood, boys have nearly twice the risk of developing asthma over girls [3, 4]. During adulthood there is a shift to a female predominance, which affects mainly non-atopic asthma [5] (Table 2). In the elderly, the gender-related differences decrease [6].

**Table 2:** Excerpt of studies on asthma epidemiology (ref 5, 6, 8), asthma symptoms (female sex hormones: ref. 33, 38, 40, 45; gender specific: ref. 52, 53) and asthma therapy (ref 61, 62)

Trial design	Results	Reference
5128 subjects Cohort study	Asthma incidence higher in women than men; female predominance stronger in non-sensitized adults	[5]
1226 asthmatic patients Cross-sectional survey	Younger women have lower quality of life and less asthma control than men	[6]
8607 subjects Cohort study	Obesity and asthma are correlated in 6–7 year old children but not in 13–14 year old teenagers	[8]
571 women Population-based cohort study	Variation of bronchial hyperreactivity during menstruation due to hormonal influences	[33]
2322 women Population-based cohort study	The odds of new onset asthma are increased in early postmenopausal women	[38]
2206 women Population-based cohort study	Hormone replacement therapy and overweight increase the risk of asthma	[40]
1438 women Population-based cohort study	Lung function decline is more rapid among post-menopausal women; respiratory health often deteriorates during reproductive aging	[45]
1248 children Population-based study; Secondary analysis	Girls with asthma have higher physical tobacco dependence scores compared to girls without asthma	[52]
3700 non-asthmatics 746 asthmatics Observational cohort study	Asthma is associated with increased risk of new onset chronic migraine; higher risk with higher number of respiratory symptoms	[53]
122 asthmatics Population-based study	No effect of inhaled corticosteroids on the decline of lung function in women compared to men	[61]
194 asthmatics Randomized, controlled trial	Montelukast decreased the risk of worsened asthma with greater benefit in young boys and older girls	[62]

In childhood, obesity, regardless of physical fitness, is associated with higher asthma prevalence and morbidity in girls, but not in boys [7]. In girls older than 11 years and women, asthma is five to seven times more common in obese people compared to those of normal weight [8, 9]. A meta-analysis showed an increased incidence of asthma in adipose, and especially in obese women [10]. In addition, pathophysiological abnormalities can be observed: blood eosinophilia seems to be more prominent in asthmatic girls [11], but in adipose asthmatic girls a higher prevalence of non-eosinophilic asthma (60.0%) compared to corresponding boys (30.8%) is the case [12].

Severe asthma affects primarily boys before and at school entry age as well as women around the time of  menopause [13]. Women also develop “corticosteroid-resistant” or difficult-to-treat asthma, more often than men [14].

There are also differences in the age-standardized mortality rates, with asthma affecting more women than men (1,37/100.000 compared to 1,16/100.000) [15]. Women (over 65 years) show a 44% higher asthma mortality than men. Black women in the US show the highest mortality rates due to asthma [16].

## Pathophysiology: Role of sex hormones and their receptors

**Fig. 1:**
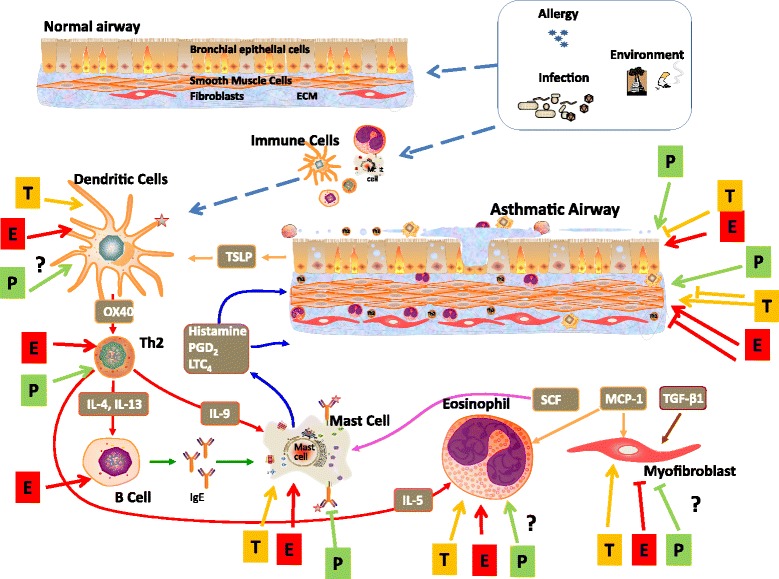
Sex steroid effects on bronchial asthma. It is recognized that asthma is a multifactorial disease involving the effects of allergic, infectious and environmental triggers on both the immune system and structural cells of the bronchial airway. Overall, inflammation drives structural and functional airway obstruction leading to epithelial thickening, increased mucus production, proliferation of epithelial, smooth muscle and fibroblast cells, remodelling of the extracellular matrix and overall airway hyperreactivity and fibrosis. Here, studies to-date suggest complex effects of oestrogen vs. progesterone vs. testosterone on relevant cell types, involving both cooperative vs. opposing effects of the different sex steroids within a cell type, but not necessarily across cell types. For example, dendritic cells, mast cells, CD4+ T lymphocytes (Th2), and eosinophils are particularly important. The effects of oestrogen (E), progesterone (P), or testosterone (T) on these immune cells can vary substantially, particularly in the context of concentration, timing and duration [19]

Oestrogen receptors are found on numerous immunoregulatory cells, and oestrogen influences immunological responses in the direction of allergy development [14]. Allergic sensitization — as demonstrated at least in animal models — is favoured not only by endogenous oestrogens but also by xeno-oestrogens from environmental pollutants such as bisphenol A and phthalates [17]. The effects of sex hormones on asthma symptoms and progression are complex and seem to be particularly associated with the fluctuation dynamics of the hormonal levels [18]. The known pathophysiological effects of sex hormones on asthma are shown in Fig. 1 [19].

Testosterone and its metabolites contribute to the physiological balance between autoimmunity and protective immunity by maintaining regulatory T cells. Testosterone has immunosuppressive effects and is probably also protective against immuno-inflammatory processes that trigger asthma [20]. This notion is supported by recent animal studies showing that male — but not female — mice were protected from allergic airway inflammation [21]. The protective effect was derived from androgen-receptor-mediated inhibition of innate immune cells type 2 (ILC2) that are key players of type 2 inflammatory responses [21].

In men, asthma morbidity remains relatively stable from puberty to the age-related drop in serum testosterone levels, which subsequently increases the risk of asthmatic impairment [22]. Previous observations provide evidence for an improvement in asthma symptoms under testosterone intake in women [23]. Testosterone thus appears to have asthma-suppressive effects, and the less virilising dehydroepiandrosterone (DHEA) as a sulphate (DHEAS), could be helpful in the therapy of premenstrual and severe asthma. Monitoring of DHEA serum level and substitution at deficient levels could therefore be considered for unstable asthmatic patients. However, further studies are necessary [24, 25].

## Symptoms in relation to female sex hormones

The clinical manifestation of asthma is different between women and men [26]. Women report more pronounced symptoms, which seem to change with the various life stages such as menstruation, pregnancy and menopause and in association with female sex hormone levels [2]. These hormones cause differences in the clinical manifestation of asthma. Thus, oestrogen promotes bronchial hyperreactivity, and both FEV1 and exhaled nitric oxide (NO) show a cycle-dependent course [27]. Twenty to forty percent (20–40%) of premenopausal women suffer from pre- or peri-menstrual asthma (PMA) and experience an exacerbation in the week preceding menstruation [18, 28], based on increased inflammation in the bronchi. This effect seems to be mediated by progesterone rather than by oestrogen [28]. PMA is less likely to be associated with atopy, but more frequently with aspirin sensitivity and poorer pulmonary function [29]. Currently the main cause for PMA is considered to be the dynamics or fluctuation rather than the absolute hormone levels, particularly of oestrogen [18, 30].

The value of hormone (substitution) therapy in women with existing asthma, and possibly also for the treatment of asthma, has so far been critically assessed and requires further studies [31]. Clinical observations and therapeutic trials showed positive effects of hormone therapy on asthma symptoms in severe perimenstrual asthma [18, 32], while both positive [33, 34] and adverse effects [35] were observed in milder and stable asthma (Table 2). It seems that the effects of oestrogen and progesterone on asthma cannot be described by a simple dose-response relationship [30].

During pregnancy, asthma can change its manifestation [36]. About one-third of women show improved asthma symptoms, one third show no change, and one third show deterioration. Asthma symptoms, which are difficult to control before pregnancy, can (but do not need to) increase even more during pregnancy. Earlier evidence that the extent of asthma symptoms is influenced by the sex of the child, with female foetuses being more frequently associated with worse asthma control, has not been confirmed [37].

In menopausal women, the risk of emergence of (mostly non-allergic) asthma generally increases [38, 39]. At the same time, oestrogen substitution with a body mass index (BMI) <30 seems to correlate with an increased risk. However, data on this topic are incongruent [40–44]. Irrespective of the presence of asthma, current data show a disproportionate loss of pulmonary function in peri/postmenopausal women compared to the age-associated decrease. The forced vital capacity (FVC) is more affected than the forced expiratory volume in one second (FEV1), which indicates a restrictive component. Oestrogen-deficit associated osteoporosis, leading to height reduction of the thoracic spine, seems to be partially involved in this restrictive reduction of pulmonary function [45] (Table 2). These changes in pulmonary function could contribute to clinically perceived asthma deterioration in menopausal women [45].

## Gender-specific symptoms

Women are more likely to suffer from coughing and wheezing, particularly at a young age, and the age-dependent decrease in bronchial hyperreactivity is less pronounced than in men. In contrast, men report symptoms during the night more frequently [46].

Men with non-allergic “intrinsic asthma” statistically display higher markers of eosinophilic airway inflammation and suffer more often from nasal polyposis than women [47]. There are different hypotheses explaining the gender-specific differences in asthma symptoms. Thus, a different perception of bronchial obstruction could be present in women compared to men. This hypothesis is supported by a consistently higher indication of dyspnoea by women compared to men, referring to the same percentage of FEV1, regardless of whether it is a small or large limitation of lung function in absolute terms [48]. Further hypotheses regarding the different symptoms between men and women take into account the lower inspiratory muscle strength as well as increased bronchial hyperreactivity in women compared to men. In dealing with their inhalation devices, women make mistakes more often [49].

Another gender difference relates to cigarette smoke, as women are more susceptible to cigarette smoke than men [50, 51]. Noticeably, girls with asthma who begin to smoke develop physical tobacco dependency much faster than girls without asthma. These differences do not seem to exist in boys [52] (Table 2).

In a study with migraine patients (gender distribution in migraine about 3:1 to the disadvantage of the female gender) the group suffering from asthma showed twice the risk of transition from episodic to chronic migraine, compared to non-asthmatics. This was statistically highly significant in the subgroup of patients with severe asthma (aOR 3,3) [19]. Thus, a similar correlation of disease severity can be observed to that of migraine patients with depression as comorbidity. The described migraine- asthma- correlation predominantly affects girls and women [19, 53].

An essential differential diagnosis of asthma — especially with unstable symptomatology — is a “vocal cord dysfunction (VCD)” or “inducible laryngeal obstruction (ILO)” [54]. In women and girls this dysfunction occurs about 4 to 5 times more frequently than in men and boys, and often leads to unnecessary, long-term adverse effects of therapeutic interventions (especially high dosed administration of systemic glucocorticosteroids) [55]. Further diagnostic and therapeutic difficulties can arise from the fact that about half of the VCD patients suffer from both classical (often severe) asthma and a vocal cord dysfunction.

Furthermore, episodes of functional, psycho-vegetatively induced hyperinflation of the lungs due to a shift of the physiological inspiration/expiration proportion seem to be more frequently observed in girls and women, which are often misinterpreted as symptoms of asthma ([56] first-hand experience of corresponding author). These episodes often lead to unnecessary and ultimately unsuccessful medication-based asthma treatment. In this case, as with VCD, breathing therapies are primarily useful, if necessary, supplemented by psychotherapeutic intervention [55].

In summary, women are more likely to have specific asthma symptoms, such as restriction of activity and shortness of breath, and they have a lower asthma-related quality of life [26, 57].

## Therapy of bronchial asthma: Gender aspects

Asthma is frequently underdiagnosed in women, and asthmatic women are less likely to receive therapy compared to asthmatic men (across all age groups) [46]. This observation was recently also reported in adolescent athletes [58]. Instead of topical steroids, women receive psychopharmaceuticals more frequently than men [46]. Women are more likely to visit their doctor unscheduled and they need emergency medicine more frequently than men [49]. It seems that the female gender is an independent risk factor for severe asthma exacerbation [49]. Despite improved lung function and less hypercapnia, emergency hospitalization is more common in women and they need longer hospital stays than men [59]. However, men have a lower therapeutic adherence in the application of their asthma therapy than women [49, 60].

For symptomatic asthma medication, such as ß2-sympathomimetics, there are no large studies showing a different effect on women or men. Concerning inhaled steroids, there are indications that in the case of “native asthmatics”, who have never been smokers, the increase of FEV1 in relation to vital capacity is significantly higher in men than in women [61] (Table 2).

Concerning the leukotriene antagonist Montelukast, it has been shown that asthma symptoms significantly improved in treated boys 2 to 9 years of age, but not in girls in the same age group. In the age group of 10- to 14-year-olds, the girls showed a much better response in comparison to the boys [62]. So the take-home message can be: Montelukast is effective in very young boys and somewhat older girls.

For biologicals such as omalizumab and mepolizumab, no prospectively collected gender-specific data are available on the therapy of asthma. For omalizumab, retrospective analyses have been published on the therapy of severe persistent asthma as well as chronic treatment-resistant urticaria, which showed no difference in the therapeutic response of women and men [63, 64].

## Summary

### What we know from epidemiology

- Childhood: boys have twice the risk of developing asthma
- Adulthood: shift to a female predominance

### What we assume based on animal studies and human intervention studies

- Female sex hormones and their receptors favour asthma development
- Male sex hormones and their receptors have a protective effect

### What we can report about gender-specific symptoms

#### Female gender

- Pronounced asthma symptoms subject to menstruation, pregnancy, menopause
- Perimenstrual asthma seems to be caused/affected by dynamic changes of oestrogen levels rather than by absolute levels
- Higher susceptibility to cigarette smoke-, migraine-, and VCD-related asthma or asthma-like symptoms
- Lower asthma-related quality of life

#### Male gender

- Pronounced age-dependent decrease in bronchial hyperreactivity
- Suffer more often from nocturnal symptoms and nasal polyps
- Show higher markers of eosinophilic airway inflammation

### What we can report about gender aspects in therapy

#### Female gender

- Asthma often underdiagnosed
- Risk factor for asthma exacerbations
- Higher responsiveness to leukotriene antagonists during puberty

#### Male gender

- Lower therapeutic adherence
- Higher responsiveness to inhaled steroids and leukotriene antagonists (the latter only during childhood)

## Conclusion and future perspectives

Different susceptibility to asthma does exist in males and females, with increased asthma prevalence and severity in adult women. However, the role of male and female sex hormones in asthma pathogenesis is not entirely elucidated. These gender differences in asthma exemplify that distinguishing different asthma phenotypes is a complex process, which should not be replaced by simple endotyping algorithms using few molecular or cellular parameters only.

## Abbreviations

DHEA: Dehydroepiandrosterone
FEV: Forced expiratory volume
FVC: Forced vital capacity
ILO: Inducible laryngeal obstruction
PMA: Pre- or perimenstrual asthma
VCD: Vocal cord dysfunction

## References

[1] Global Initiative for Asthma (GINA). http://www.ginasthma.org/2017-gina-report-global-strategy-for-asthma-management-and-prevention/. 2017.

[2] Fuseini H, Newcomb DC (2017). Mechanisms driving gender differences in asthma. Curr Allergy Asthma Rep.

[3] Carey MA, Card JW, Voltz JW, Arbes SJ, Germolec DR, Korach KS (2007). It's all about sex: gender, lung development and lung disease. Trends Endocrinol Metab.

[4] Jensen-Jarolim E, Untersmayr E (2008). Gender-medicine aspects in allergology. Allergy.

[5] Hansen S, Probst-Hensch N, Keidel D, Dratva J, Bettschart R, Pons M (2015). Gender differences in adult-onset asthma: results from the Swiss SAPALDIA cohort study. Eur Respir J.

[6] Lisspers K, Stallberg B, Janson C, Johansson G, Svardsudd K (2013). Sex-differences in quality of life and asthma control in Swedish asthma patients. J Asthma.

[7] Lu KD, Billimek J, Bar-Yoseph R, Radom-Aizik S, Cooper DM, Anton-Culver H. Sex differences in the relationship between fitness and obesity on risk for asthma in adolescents. J Pediatr. 2016;176:36–42. 10.1016/j.jpeds.2016.05.050 PMC5003726 27318375

[8] Alvarez Zallo N, Aguinaga-Ontoso I, Alvarez-Alvarez I, Guillen-Grima F (2017). Azcona san Julian C. The influence of gender and atopy in the relationship between obesity and asthma in childhood. Allergol Immunopathol.

[9] Weiss ST, Shore S (2004). Obesity and asthma: directions for research. Am J Respir Crit Care Med.

[10] Beuther DA, Weiss ST, Sutherland ER (2006). Obesity and asthma. Am J Respir Crit Care Med.

[11] Rubner F, Jackson D, Tisler C, Rajamanickam V, Gern J, Lamanske R. Relationships among eosinophils, asthma, and sex in a high-risk birth cohort. The Journal of allergy and clinical immunology. 2015;135(2 Suppl. 1):AB228. doi:10.1016/j.jaci.2014.12.1680.

[12] Jensen ME, Gibson PG, Collins CE, Wood LG (2013). Airway and systemic inflammation in obese children with asthma. Eur Respir J.

[13] Zein JG, Udeh BL, Teague WG, Koroukian SM, Schlitz NK, Bleecker ER (2016). Impact of age and sex on outcomes and hospital cost of acute asthma in the United States, 2011-2012. PLoS One.

[14] Keselman A, Heller N (2015). Estrogen signaling modulates allergic inflammation and contributes to sex differences in asthma. Front Immunol.

[15] DesMeules M, Manuel D, Cho R (2004). Mortality: life and health expectancy of Canadian women. BMC women’s health.

[16] Kynyk JA, Mastronarde JG, McCallister JW (2011). Asthma, the sex difference. Curr Opin Pulm Med.

[17] Bonds RS, Midoro-Horiuti T (2013). Estrogen effects in allergy and asthma. Curr Opin Allergy Clin Immunol.

[18] Graziottin A, Serafini A (2016). Perimenstrual asthma: from pathophysiology to treatment strategies. Multidisciplinary respiratory medicine.

[19] Sathish V, Martin YN, Prakash YS (2015). Sex steroid signaling: implications for lung diseases. Pharmacol Ther.

[20] Canguven O, Albayrak S (2011). Do low testosterone levels contribute to the pathogenesis of asthma?. Med Hypotheses.

[21] Laffont S, Blanquart E, Savignac M, Cenac C, Laverny G, Metzger D (2017). Androgen signaling negatively controls group 2 innate lymphoid cells. J Exp Med.

[22] Zannolli R, Morgese G (1997). Does puberty interfere with asthma?. Med Hypotheses.

[23] Wulfsohn NL, Politzer WM, Henrico JS (1964). Testosterone therapy in bronchial asthma. S Afr Med J.

[24] Choi IS (2011). Gender-specific asthma treatment. Allergy, Asthma Immunol Res.

[25] Wenzel SE, Robinson CB, Leonard JM, Panettieri RA (2010). Nebulized dehydroepiandrosterone-3-sulfate improves asthma control in the moderate-to-severe asthma results of a 6-week, randomized, double-blind, placebo-controlled study. Allergy Asthma Proc.

[26] Pignataro FS, Bonini M, Forgione A, Melandri S, Usmani OS (2017). Asthma and gender: the female lung. Pharmacol Res.

[27] Clark NM, Valerio MA, Gong ZM (2008). Self-regulation and women with asthma. Curr Opin Allergy Clin Immunol.

[28] Saxena P, Shukla S, Kriplani K, Agarwal R (2007). A review on premenstrual asthma: an Underrecognised entity. Journal of Internal Medicine of India.

[29] Farha S, Asosingh K, Laskowski D, Hammel J, Dweik RA, Wiedemann HP (2009). Effects of the menstrual cycle on lung function variables in women with asthma. Am J Respir Crit Care Med.

[30] Baldacara RP, Silva I (2017). Association between asthma and female sex hormones. Sao Paulo medical journal = Revista paulista de medicina.

[31] Murray RD, New JP, Barber PV, Shalet SM (1999). Gonadotrophin-releasing hormone analogues: a novel treatment for premenstrual asthma. Eur Respir J.

[32] Derimanov GS, Oppenheimer J (1998). Exacerbation of premenstrual asthma caused by an oral contraceptive. Ann Allergy Asthma Immunol.

[33] Dratva J, Schindler C, Curjuric I, Stolz D, Macsali F, Gomez FR (2010). Perimenstrual increase in bronchial hyperreactivity in premenopausal women: results from the population-based SAPALDIA 2 cohort. J Allergy Clin Immunol.

[34] Forbes L, Jarvis D, Burney P (1999). Do hormonal contraceptives influence asthma severity?. Eur Respir J.

[35] Pforte A (2008). Geschlechtsspezifische Differenzen bei Lungenerkrankungen. Pneumologie.

[36] Firoozi F, Ducharme FM, Lemiere C, Beauchesne MF, Perreault S, Forget A (2009). Effect of fetal gender on maternal asthma exacerbations in pregnant asthmatic women. Respir Med.

[37] Tamasi L, Horvath I, Bohacs A, Muller V, Losonczy G, Schatz M (2011). Asthma in pregnancy--immunological changes and clinical management. Respir Med.

[38] Triebner K, Johannessen A, Puggini L, Benediktsdottir B, Bertelsen RJ, Bifulco E (2016). Menopause as a predictor of new-onset asthma: a longitudinal northern European population study. J Allergy Clin Immunol.

[39] van den Berge M, Heijink HI, van Oosterhout AJ, Postma DS (2009). The role of female sex hormones in the development and severity of allergic and non-allergic asthma. Clin Exp Allergy.

[40] Gomez Real F, Svanes C, Bjornsson EH, Franklin KA, Gislason D, Gislason T (2006). Hormone replacement therapy, body mass index and asthma in perimenopausal women: a cross sectional survey. Thorax.

[41] Haggerty CL, Ness RB, Kelsey S, Waterer GW. The impact of estrogen and progesterone on asthma. Ann Allergy Asthma Immunol. 2003;90(3):284–91; quiz 91–3, 347. 10.1016/S1081-1206(10)61794-2 12669890

[42] Myers JR, Sherman CB (1994). Should supplemental estrogens be used as steroid-sparing agents in asthmatic women?. Chest.

[43] Townsend EA, Miller VM, Prakash YS (2012). Sex differences and sex steroids in lung health and disease. Endocr Rev.

[44] Zierau O, Zenclussen AC, Jensen F (2012). Role of female sex hormones, estradiol and progesterone, in mast cell behavior. Front Immunol.

[45] Triebner K, Matulonga B, Johannessen A, Suske S, Benediktsdottir B, Demoly P, et al. Menopause is associated with accelerated lung function decline. Am J Respir Crit Care Med. 2016; 10.1164/rccm.201605-0968OC 27907454

[46] Grohe C, Oertelt-Prigione S, Regitz-Zagrosek V (2012). Sex and gender differences in pulmonary diseases. Sex and gender aspects in clinical medicine.

[47] Amelink M, Nijs SD, Groot CD, S Lone-Laktif, Reinartz S, Fokkens W, et al. Nasal Polyposis Identifies an at Risk Phenotype among PAtients with adult-onset Asthma. AJRCCM. 2011(183.1):Abstract A3724.

[48] Becklake MR, Kauffmann F (1999). Gender differences in airway behaviour over the human life span. Thorax.

[49] McCallister JW, Mastronarde JG (2008). Sex differences in asthma. J Asthma.

[50] Kamil F, Pinzon I, Foreman MG (2013). Sex and race factors in early-onset COPD. Curr Opin Pulm Med.

[51] Sorheim IC, Johannessen A, Gulsvik A, Bakke PS, Silverman EK, DeMeo DL (2010). Gender differences in COPD: are women more susceptible to smoking effects than men?. Thorax.

[52] Guo SE, Ratner PA, Okoli CT, Johnson JL (2014). The gender-specific association between asthma and the need to smoke tobacco. Heart Lung.

[53] Martin VT, Fanning KM, Serrano D, Buse DC, Reed ML, Lipton RB (2016). Asthma is a risk factor for new onset chronic migraine: results from the American migraine prevalence and prevention study. Headache.

[54] Balkissoon R, Kenn K (2012). Asthma: vocal cord dysfunction (VCD) and other dysfunctional breathing disorders. Semin Respir Crit Care Med.

[55] Kenn K, Hess MM (2008). Vocal cord dysfunction: an important differential diagnosis of bronchial asthma. Deutsches Arzteblatt international.

[56] Zein JG, Erzurum SC (2015). Asthma is different in women. Curr Allergy Asthma Rep.

[57] McCallister JW, Holbrook JT, Wei CY, Parsons JP, Benninger CG, Dixon AE (2013). Sex differences in asthma symptom profiles and control in the American Lung Association asthma clinical research centers. Respir Med.

[58] Romberg K, Tufvesson E, Bjermer L (2017). Sex differences in asthma in swimmers and tennis players. Ann Allergy Asthma Immunol.

[59] Koper I (2015). Gender-specific differences in obstructive lung diseases. Pneumologie.

[60] Lindner PS, Lindner AJ (2014). Gender differences in asthma inhaler compliance. Conn Med.

[61] Dijkstra A, Vonk JM, Jongepier H, Koppelman GH, Schouten JP, ten Hacken NH (2006). Lung function decline in asthma: association with inhaled corticosteroids, smoking and sex. Thorax.

[62] Johnston NW, Mandhane PJ, Dai J, Duncan JM, Greene JM, Lambert K (2007). Attenuation of the September epidemic of asthma exacerbations in children: a randomized, controlled trial of montelukast added to usual therapy. Pediatrics.

[63] Bousquet J, Cabrera P, Berkman N, Buhl R, Holgate S, Wenzel S (2005). The effect of treatment with omalizumab, an anti-IgE antibody, on asthma exacerbations and emergency medical visits in patients with severe persistent asthma. Allergy.

[64] Viswanathan RK, Moss MH, Mathur SK (2013). Retrospective analysis of the efficacy of omalizumab in chronic refractory urticaria. Allergy Asthma Proc.

